# Comparison of clinical features between immune-related sclerosing cholangitis and hepatitis

**DOI:** 10.1007/s10637-021-01136-z

**Published:** 2021-05-28

**Authors:** Masaki Takinami, Akira Ono, Takanori Kawabata, Nobuaki Mamesaya, Haruki Kobayashi, Shota Omori, Kazushige Wakuda, Hirotsugu Kenmotsu, Tateaki Naito, Haruyasu Murakami, Masahiro Endo, Yoshio Kiyohara, Hirofumi Yasui, Masashi Niwakawa, Toshiaki Takahashi

**Affiliations:** 1 Division of Thoracic Oncology, Shizuoka Cancer Center, 1007 Shimonagakubo, Nagaizumi, Sunto-gun, Shizuoka, 411-8777 Japan; 2 Division of Gastrointestinal Oncology, Shizuoka Cancer Center, 1007 Shimonagakubo, Nagaizumi, Sunto-gun, Shizuoka, 411-8777 Japan; 3 Clinical Research Center, Shizuoka Cancer Center, 1007 Shimonagakubo, Nagaizumi, Sunto-gun, Shizuoka, 411-8777 Japan; 4 Division of Diagnostic Radiology, Shizuoka Cancer Center, 1007 Shimonagakubo, Nagaizumi, Sunto-gun, Shizuoka, 411-8777 Japan; 5 Division of Dermatology, Shizuoka Cancer Center, 1007 Shimonagakubo, Nagaizumi, Sunto-gun, Shizuoka, 411-8777 Japan; 6 Division of Urology, Shizuoka Cancer Center, 1007 Shimonagakubo, Nagaizumi, Sunto-gun, Shizuoka, 411-8777 Japan

**Keywords:** Immune-related adverse events, Drug-induced liver injury, Secondary sclerosing cholangitis, Regulatory T cells

## Abstract

*Background* Immune-related hepatotoxicity is often regarded as immune-related hepatitis (irHepatitis) despite including immune-related sclerosing cholangitis (irSC). This study examined the clinical differences between irSC and irHepatitis. *Methods* A single-center retrospective study of 530 consecutive patients who received immunotherapy between August 2014 and April 2020 was performed. IrSC and irHepatitis were respectively defined as the radiological presence and absence of bile duct dilation and wall thickness. *Results* Forty-one patients (7.7%) developed immune-related hepatotoxicity. A CT scan was performed on 12 patients, including 11 of 12 with ≥ grade 3 aminotransferase elevations. IrSC and irHepatitis were diagnosed in 4 (0.8%) and 8 (1.5%) patients, respectively. All the irSC patients had been treated with anti-PD-1. IrHepatitis was more common among patients receiving anti-CTLA-4 than among those receiving anti-PD-1/PD-L1 inhibitors (14%, 7/50 vs. 0.2%, 1/480, *P* < 0.001). A ≥ grade 2 alkaline phosphatase (ALP) elevation resulting in a cholestatic pattern was seen in all 4 irSC patients. Among the irSC patients, 3 (3/4, 75%) developed ≥ grade 3 aminotransferases elevation. The median duration from the start of immunotherapy until ≥ grade 2 liver enzymes elevation was 257 and 55.5 days in irSC and irHepatitis patients. The median times for progression from grade 2 to 3 liver enzyme elevation were 17.5 and 0 days, respectively. *Conclusions* IrSC and irHepatitis have different characteristics in the class of immune checkpoint inhibitor and onset pattern. Radiological examination for the diagnosis of irSC should be considered for patients with ≥ grade 2 ALP elevation resulting in a cholestatic pattern. (Registration number J2020-36, Date of registration June 3, 2020)

## Introduction

Immune-related hepatotoxicity is a major adverse event associated with the use of immune checkpoint inhibitors (ICIs), developing in up to 16% of patients who receive immunotherapy [1].

Drug-induced hepatotoxicity is basically divided into hepatocellular, cholestatic, and mixed patterns based on the R ratio, which is calculated from the alanine aminotransferase (ALT) and alkaline phosphatase (ALP) levels [2]. These three patterns are also observed in patients receiving ICIs [3]. However, the guidelines for immunotherapy toxicities regard immune-related hepatotoxicity as immune-related hepatitis (irHepatitis) [4, 5].

Immune-related sclerosing cholangitis (irSC) has been recognized as a secondary sclerosing cholangitis induced by ICIs since 2017 [6, 7]. The clinical manifestations of irSC are bile duct dilatations and wall thickness developing after the start of immunotherapy [7]. These imaging findings are common to primary sclerosing cholangitis and immunoglobulin G4-related sclerosing cholangitis [8, 9]. Thus, bile duct imaging is an important diagnostic step for the diagnosis of irSC. In contrast, a computed tomography (CT) scan is not recommended, except for the assessment of hepatic metastases or thrombosis, in patients with immune-related hepatotoxicity,^4^ since the imaging features of hepatitis are nonspecific [10]. In addition, irHepatitis is a diagnosis of exclusion, and its differential diagnosis does not include irSC [4]. Therefore, some patients with irSC would be misdiagnosed with irHepatitis due to the lack of CT imaging. The reason for the unclear distinction between irSC and irHepatitis is that the clinical features of irSC are not well-known, because of limited reports [11]. This study aimed to elucidate the clinical features of radiologically classified irSC and irHepatitis in patients developing immune-related hepatotoxicity [4].

## Methods

### Patients and data collection

We retrospectively reviewed the data of 530 consecutive patients who received ICIs between August 2014 and April 2020 at the Shizuoka Cancer Center. Patient characteristics including age; sex; laboratory data for aspartate aminotransferase (AST), ALT, ALP, and total bilirubin; and clinical course were recorded. Elevations in liver enzymes occurring at any time after the start of immunotherapy, were evaluated according to the Common Terminology Criteria for Adverse Events (CTCAE), v.5.0 [12]. This study was approved by the Ethics Committee of our center (approval no. J2020-36).

### Immunotherapies

The patients had received the following treatments: (1) nivolumab (80 mg) plus ipilimumab (3 mg/kg) every 3 weeks for 4 doses, then nivolumab (240 mg) every 2 weeks for the treatment of melanoma or renal cell cancer; (2) ipilimumab (3 mg/kg) every 3 weeks for the treatment of melanoma; (3) nivolumab (3 mg/kg or 240 mg) every 2 weeks for the treatment of melanoma, renal cell cancer, non-small cell lung cancer, head and neck cancer, gastric cancer, or Hodgkin lymphoma; (4) pembrolizumab (200 mg) every 3 weeks for the treatment of melanoma, non-small cell lung cancer, urothelial cancer or microsatellite instability‒high solid cancer; (5) atezolizumab (1200 mg) every 3 weeks for non-small or small cell lung cancer; (6) durvalumab (10 mg/kg) every 2 weeks for non-small cell lung cancer; and (7) avelumab (10 mg/kg) every 2 weeks for Merkel cell cancer.

### Definitions

Hepatotoxicity was defined as an AST or ALT or ALP elevation of ≥ grade 2. Immune-related hepatotoxicity at onset was assessed with a score of 6 to 8 interpreted as having a “probable” causality and a score of greater than 8 interpreted as having a “highly probable” causality according to the RUCAM scale, which excludes viral hepatitis, alcohol, hypotension, shock or ischemia, complications from underlying disease, and serologic factors as possible causes [2]. Patients with a clinical course suggesting liver metastasis as a secondary cause of hepatotoxicity were also excluded [13].

**Fig. 1:**
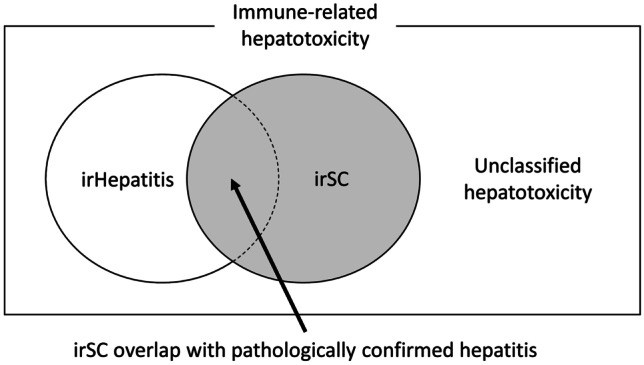
Types of immune-related hepatotoxicity

Figure 1 shows the radiological classification of immune-related hepatotoxicity. Among patients with immune-related hepatotoxicity, irSC was radiologically defined as the development of bile duct dilation and wall thickness after the initiation of ICI. Bile duct dilation was defined as the coexistence of bile duct stenosis without obstruction, and wall thickness was defined as more than double that before immunotherapy. IrHepatitis was radiologically defined as the absence of these features. Therefore, irSC overlapping with histologically confirmed hepatitis was radiologically diagnosed irSC, and patients who did not undergo a CT scan were diagnosed as having unclassified hepatotoxicity.

### Outcome analysis

The fisher’s exact test and the Mann–Whitney *U* test were used to compare the proportion of categorical variables and the median of continuous variables between groups. The equality of the cumulative incidence was evaluated using Gray’s test. A logistic regression analysis was used to identify risk factors for ≥ grade 3 hepatotoxicity using factors with a *P*-value < 0.05 in a univariable analysis. A *P*- value < 0.05 was considered to be significant, and all calculations were performed using EZR ver.1.37 [14].

## Results

### Patient flow diagram and patient characteristics

The characteristics of all the patients who received ICIs are summarized in Table 1. The median age was 68.5 years. Males were predominant, with a male:female ratio of 2:1. Non-small cell lung cancer and melanoma were predominant. Anti-CTLA-4 inhibitor was used to treat 50 (9.4%) patients.

**Table 1:** Characteristics of patients treated with immune checkpoint inhibitors

	N = 530 (%)
Age, median (IQR), years	68.5 (61.0–74.0)
Sex	
Male	350 (66%)
Female	180 (34%)
Malignancy	
Non-small cell lung cancer	348 (66%)
Melanoma	98 (18%)
Renal cell cancer	27 (5%)
Head and neck cancer	18 (3%)
Others	39 (7%)
ICI	
Anti-CTLA-4 ± anti-PD1	50 (9%)
Anti-PD-1/PD-L1 alone	480 (91%)
PD-L1 expression (> 1%)	170/234 (73%)
Number of ICI infusions	
1–2	145 (27%)
3–10	258 (49%)
More than 11	127 (24%)

*ALP* alkaline phosphatase, *ALT* alanine aminotransferase, *AST* aspartate aminotransferase, *ICI* immune checkpoint inhibitor, *IQR* interquartile range, *T-Bil* total bilirubin

**Fig. 2:**
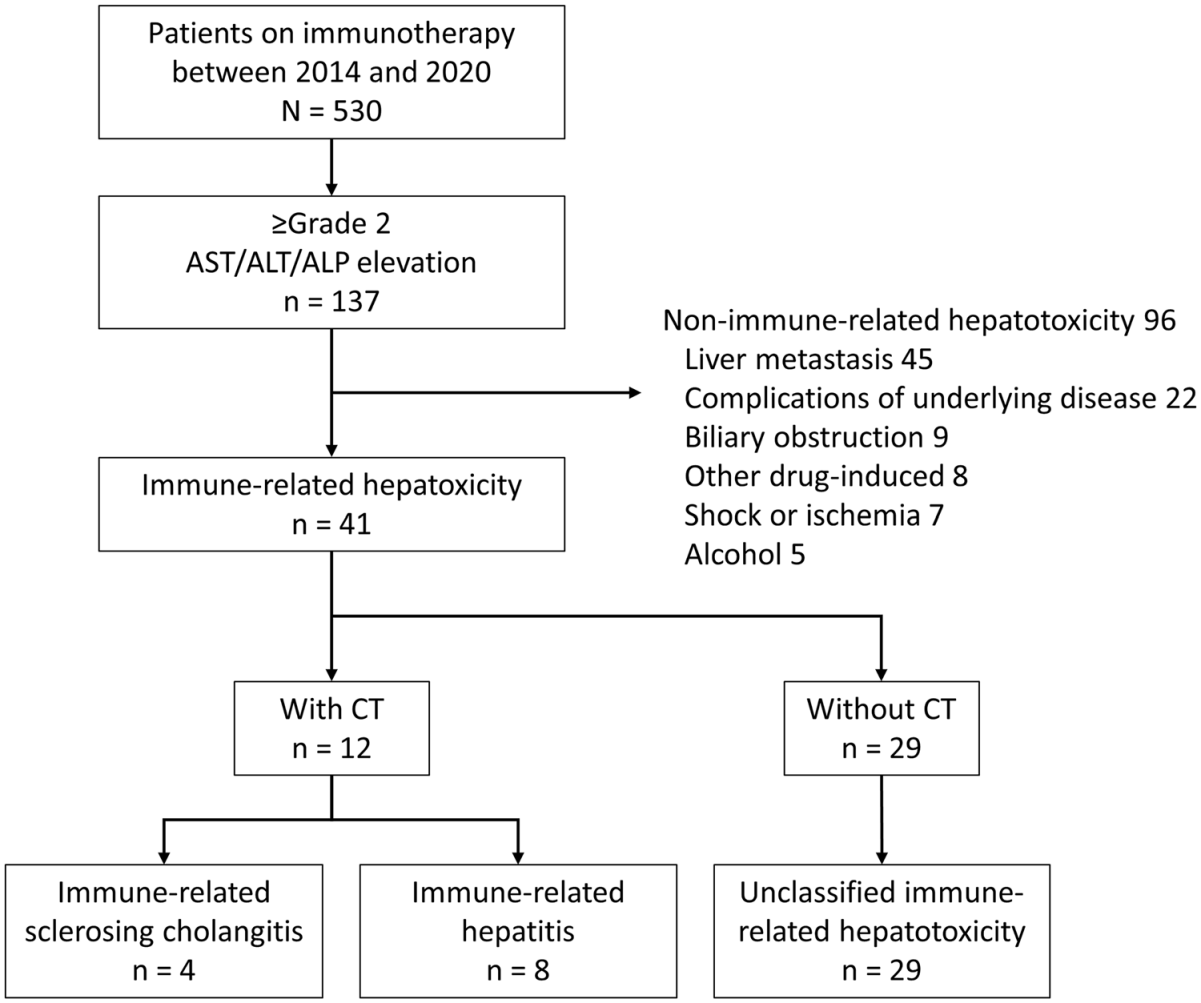
A flow-diagram of patients with hepatotoxicity after the initiation of immune checkpoint inhibitor therapy

Figure 2 shows a patient flow diagram. Overall, 137 patients (26%) had ≥ grade 2 AST, ALT, or ALP elevations, and 41 (7.7%) developed immune-related hepatotoxicity. A CT scan was performed in 12 patients: 1 of the 28 (3.6%) patients with a ≤ grade 2 AST or ALT elevation, and 11 of the 13 (85%) patients with a ≥ grade 3 AST or ALT elevation. Of these patients, 4 (0.8%) were radiologically diagnosed as having irSC, and 8 (1.5%) were radiologically diagnosed as having irHepatitis. Of 29 patients without CT imaging, 27 (93%) showed a grade 2 AST or ALT elevation and 29 (100%) improved without requiring corticosteroids.

### Clinical features of immune-related sclerosing cholangitis and hepatitis

Table 2 shows the clinical features of the patients with irSC and irHepatitis. All 4 patients with irSC were treated with anti-PD-1 inhibitors and had ≥ grade 2 ALP elevations resulting in a cholestatic pattern at onset. Three irSC patients (3/4, 75%) subsequently developed grade 3 AST or ALT elevations. Liver biopsies were performed in 2 irSC patients, revealing a high CD8^+^/CD4^+^ cell ratio around the bile ducts in both patients and centrilobular zonal necrosis in 1 patient. Bile duct biopsies obtained from 2 irSC patients showed severe inflammatory changes, which were nonspecific for irSC.

**Table 2:** Principal characteristics of patients with immune-related sclerosing cholangitis and hepatitis

Type of injury	No	Age	Sex	Disease	Drug	Pattern of onset	ALT/ALP (at the worst ALT)	Biopsy site
irSC	1	M	81	NSCLC	Pembro	Cholestatic	419/1987	Liver & bile duct
	2	F	83	NSCLC	Pembro	Cholestatic	237/4847	Liver
	3	M	71	NSCLC	Nivo	Cholestatic	74/1613	Bile duct
	4	M	68	NSCLC	Nivo	Cholestatic	91/3117	None
irHepatitis	1	M	68	NSCLC	Pembro	Hepatocellular	580/531	None
	2	F	31	Melanoma	Ipi + Nivo	Hepatocellular	1976/323	None
	3	F	56	Melanoma	Ipi + Nivo	Hepatocellular	1293/683	None
	4	F	78	Melanoma	Ipi + Nivo	Cholestatic	215/880	None
	5	M	54	Melanoma	Ipi + Nivo	Mixed	809/1446	None
	6	F	68	Melanoma	Ipi	Mixed	425/589	None
	7	M	55	Melanoma	Ipi	Mixed	236/512	None
	8	M	54	Renal cancer	Ipi + Nivo	Hepatocellular	614/1096	None

*AST* aspartate aminotransferase, *ALT* alanine aminotransferase, *ALP* alkaline phosphatase, *Ipi* ipilimumab, *Nivo* nivolumab, *NSCLC* non-small cell lung cancer, *Pembro* pembrolizumab, *T-Bil* total bilirubin

### Comparison of clinical features

**Fig. 3:**
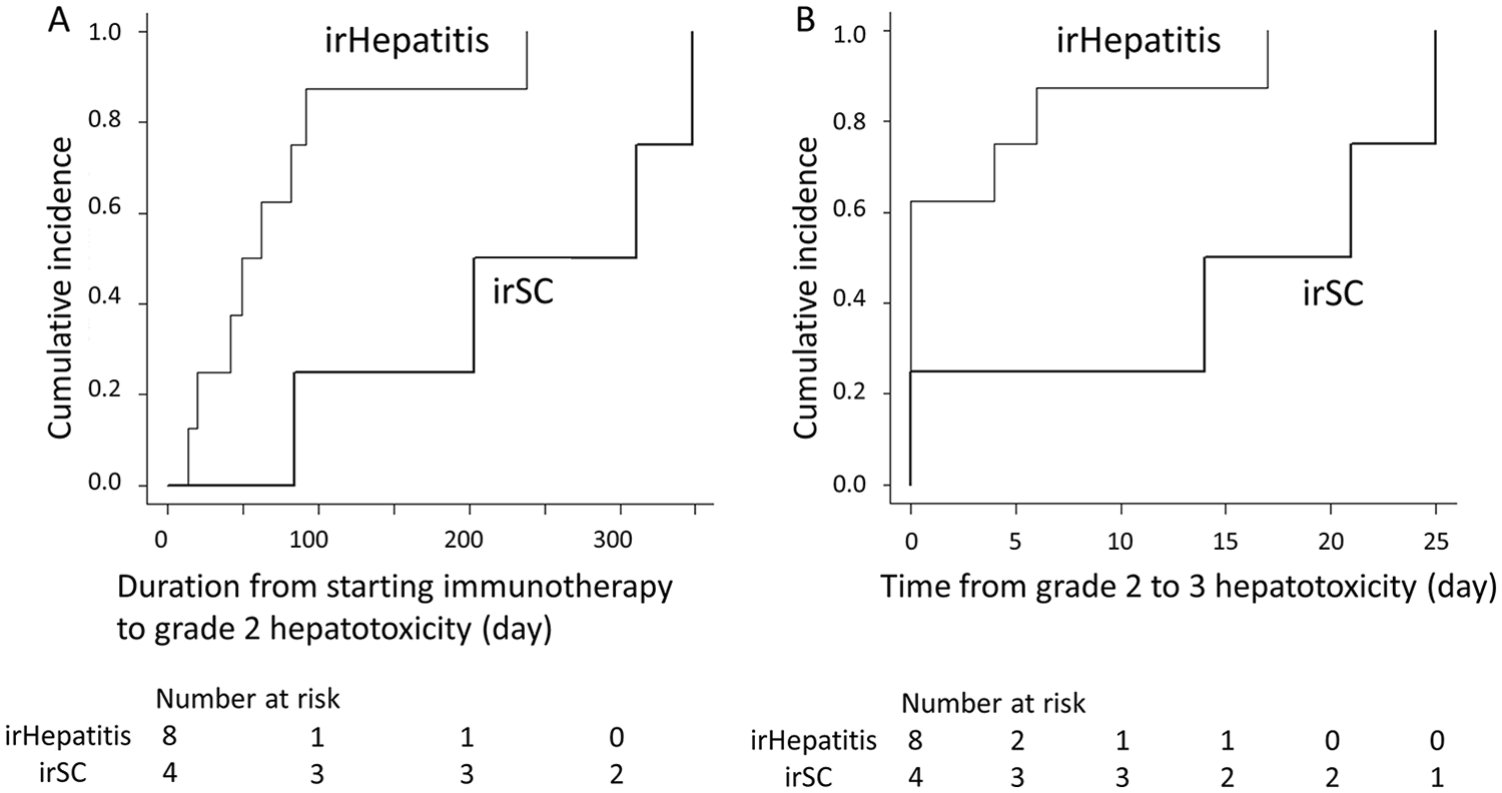
Onset pattern of hepatotoxicity. (**A**) Duration from the start of immunotherapy to the onset of a ≥ grade 2 liver enzymes elevation. (**B**) Time for the progression from a grade 2 to a grade 3 elevation in liver enzymes. The thick line shows hepatitis, and the bold line shows sclerosing cholangitis

Comparisons of the clinical features of irSC and irHepatitis are shown in Table 3 and Fig. 3. The use of anti-CTLA-4 inhibitor did not induce irSC compared with irHepatitis (0 vs. 7). A ≥ grade 2 ALP elevation resulting in a cholestatic pattern at onset was seen more often in patients with irSC than in those with irHepatitis (4 vs. 1). The median duration from the initiation of ICI treatment to a ≥ grade 2 elevation in AST, ALT, or ALP was significantly longer in the patients with irSC than in those with irHepatitis (257 vs. 55.5 days). The median time for progression a from grade 2 to a grade 3 elevation in AST, ALT, or ALP was longer among patients with irSC than among those with irHepatitis (17.5 vs. 0 days).

**Table 3:** Demographic and background features of patients with immune-related sclerosing cholangitis and hepatitis

	Immune-related hepatitis n = 8 (%)	Immune-related sclerosing cholangitis n = 4 (%)
Age, median (IQR), years	55.5 (54–68)	76 (70–82)
Sex		
Male	4 (50%)	3 (75%)
Female	4 (50%)	1 (25%)
Symptom Fever	1 (13%)	1 (25%)
Fatigue	5 (63%)	0 (0%)
Disease Non-small cell lung cancer	1 (13%)	4 (100%)
Melanoma	6 (75%)	0 (0%)
Renal cell cancer	1 (13%)	0 (0%)
Immune checkpoint inhibitor		
Anti-CTLA-4 ± anti-PD1	7 (87%)	0 (0%)
Anti-PD-1/PD-L1 alone	1 (13%)	4 (100%)
PD-L1 expression	4/5 (80%)	3/4 (75%)
Hepatotoxicity pattern at onset		
≥ Grade 3 aminotransferases	5 (63%)	1 (25%)
≥ Grade 2 alkaline phosphatase	3 (38%)	4 (100%)
Median R ratio at onset, (IQR)	4.1 (1.1–7.5)	0.6 (0.2–3.0)
Cholestatic pattern	1 (13%)	4 (100%)
Mixed pattern	3 (38%)	0 (0%)
Hepatocellular pattern	4 (50%)	0 (0%)
Worst laboratory data		
≥ Grade 3 aminotransferases	8 (100%)	3 (75%)
≥ Grade 3 alkaline phosphatase	1 (13%)	4 (100%)
Jaundice	5 (63%)	2 (50%)
Cycles of ICI infusion, median (IQR)	2 (2–3.25)	3 (2–4.3)
Days until onset, median (IQR)	55.5 (37–85)	257 (173–320)
Days until progression from Grade 2 to 3 elevation, median (IQR)	0 (0–4.5)	17.5 (11–25)
Treatment Corticosteroid	6 (75%)	1 (25%)
Mycophenolate mofetil	2 (25%)	0 (0%)
Days until resolution to Grade 1 elevation, median (IQR)	36 (25–54)	102.5 (72–134)

*ICI* immune checkpoint inhibitor, *IQR* interquartile range

### Treatment outcomes

ICI treatment was resumed in all the patients with irSC or irHepatitis. Steroid therapy was administered to 1 patient with irSC (1/4, 25%) and to 6 patients with irHepatitis (6/8, 75%). One irSC patient treated with steroid therapy showed a worsening of an intralobular bile duct stricture after an improvement in the laboratory data. The conditions of 4 irHepatitis patients resolved with steroid therapy (4/6, 67%), while the treatments of 2 patients (2/6, 33%) were switched to mycophenolic mofetil. A rechallenge with the same ICI after the resolution of the hepatotoxicity was performed in 1 irHepatitis patient, whereas switching to other ICIs was performed in 2 irHepatitis patients and 1 irSC patients. All 4 patients treated with the reintroduction of ICI did not develop immune-related hepatotoxicity again.

### Risk factors for ≥ grade 3 aminotransferases elevations

Table 4 shows the risk factors for ≥ grade 3 AST or ALT elevations. A ≥ grade 3 AST or ALT elevation was seen in a total of 13 patients: 8 with irHepatitis, 3 with irSC and 2 with unclassified hepatotoxicity. Univariate analyses showed that a female sex, melanoma, and the use of anti-CTLA-4 inhibitor were significantly correlated with ≥ grade 3 AST or ALT elevations. A logistic regression analysis revealed that the use of anti-CTLA-4 inhibitor was a risk factor.

**Table 4:** Risk factors for ≥ grade 3 aminotransferases elevations

	≤ Grade 2 elevation n = 28 (%)	≥ Grade 3 elevation n = 13 (%)	*P* value	Univariate regression		Multivariate regression	
				Odds ratio (95% CI)	*P* value	Odds ratio (95% CI)	*P* value
Age, median (IQR), years	66 (59–72)	68 (55–84)	0.81				
Sex			0.02				
Female	3 (11%)	6 (46%)		7.1 (1.4–36)	0.02	5.6 (0.88–33)	0.07
Male	25 (89%)	7 (54%)		1 (Ref)		1 (Ref)	
Disease			0.049				
Melanoma	4 (14%)	6 (46%)		5.1 (1.1–24)	0.03	0.89 (0.07–11)	0.92
Others	24 (86%)	7 (54%)		1 (Ref)		1 (Ref)	
Prior irAE	6 (21%)	2 (15%)	1.00				
Autoimmune disease	1 (4%)	0 (0%)	1.00				
ICI class							
Anti-PD-1/PD-L1	26 (93%)	6 (46%)	0.002	1 (Ref)		1 (Ref)	
Anti-CTLA-4 ± PD-1	2 (7%)	7 (54%)		15.2 (2.5–92)	0.003	13.7 (1.2–161)	0.037
PD-L1 expression	8/13 (62%)	9/10 (90%)	0.18				

*CI* confidence interval, *ICI* immune checkpoint inhibitor, *IQR* interquartile range, *irAE* immune-related adverse events, *Ref* Reference

Among all 530 patients, the incidence of irHepatitis was significantly higher among patients with melanoma than among those with other cancers (6%, 6/98 vs. 0.5%, 2/432, *P* < 0.001); the incidence of irHepatitis was also higher among patients receiving anti-CTLA-4 inhibitor than among those receiving anti-PD-1/PD-L1 inhibitors (14%, 7/50 vs. 0.2%, 1/480, *P* < 0.001). The proportion of female to male was higher among patients with melanoma compared with other cancers (50%, 49/98 vs. 33%, 131/432, *P* < 0.001).

## Discussion

This is the first report to focus on a comparison of irSC and irHepatitis. The present study showed that the use of anti-CTLA-4 inhibitor did not induce irSC, and that irSC showed a slow and gradual onset with a ≥ grade 2 ALP elevation resulting in a cholestatic pattern, compared with irHepatitis. These findings are clinically important, because a ≥ grade 2 ALP elevation resulting in a cholestatic pattern will lead to diagnostic doubt of irSC, suggesting the addition of CT scans.

Distinguishing between irSC and irHepatitis is important for predicting steroid responsiveness. The response rate for steroid therapy is reportedly only 11.5% among irSC patients [11]. Also, in the present study, an irSC case treated with a corticosteroid showed a worsening of bile duct strictures, even after an improvement in the liver enzyme levels [15]. In contrast, 98% of irHepatitis patients showed a resolution of their condition after treatment with corticosteroids or improved without requiring corticosteroids in a previous report [16].

The incidence of irHepatitis depends on the class of ICI and is higher for patients treated with ipilimumab alone (4.5%) or combination with nivolumab (13%), compared with anti-PD-1 inhibitor alone (1.8%) [1, 17]. In contrast, a review of case studies showed that none of the 31 irSC cases were associated with anti-CTLA-4 inhibitor [11]. From a histological perspective, anti-CTLA-4 inhibitor causes granulomas hepatitis with central vein endotheliitis, while anti-PD-1/PD-L1 inhibitor induces a heterogenous injury pattern without granulomatous inflammation [18, 19]. Endotheliitis is often present in acute liver allograft rejection [20], in which rejection is induced by the blockage of immunosuppressive regulatory T cells (Tregs) via the CTLA-4 pathway [21]. Anti-CTLA-4 inhibitor binds to CTLA-4 on Tregs in vivo [22], and Treg differentiation is induced by endothelial cells [23]. Anti-CTLA-4 inhibitors might modulate the Treg signaling pathway, inducing irHepatitis with endotheliitis. While PD-1/L-1 inhibitors causes inflammation with a high rate of CD8^+^/CD4^+^ cells, which is a pathological feature of immune-related adverse event in the hepatobiliary system [24]. The CD8^+^ cells associate with sclerosing cholangitis by producing interferon gamma [25]. The invasion of the CD8^+^ inflammation cells may have influenced its association with the immune-related cholangitis. Different classes of ICIs might have different mechanisms of action and be associated with the development of irSC and irHepatitis.

In the present study, irSC showed a slow and gradual onset with cholestatic pattern compared to irHepatitis. The median number of cycles until the onset of hepatotoxicity has been reported to be 5.5 in irSC patients and 1 to 3 for irHepatitis patients [11, 16]. Thus, the onset of irHepatitis is thought to be more acute than that of irSC.

Overlap is a condition in which multiple diseases coexist, such as autoimmune hepatitis and primary sclerosing cholangitis. The possibility of irHepatitis-irSC overlap should also be considered, as Stuart et al. preciously reported one case [26]. In 16 patients who underwent a liver biopsy out of 536 irHepatitis patients, more than half of them presented with bile duct injury [18]. Conversely, irHepatitis was found in 13.3% of irSC patients [11]. Although a liver biopsy can make a definitive diagnosis of irHepatitis-irSC overlap, bleeding after a liver biopsy is generally considered the major complication. Therefore, bile duct imaging can be an important step in evaluating the coexistence of irSC which indicates a poor responsiveness to corticosteroid and in determining if a liver biopsy is needed even after a diagnosis of irHepatitis.

Clinical factors predicting a risk of immune-related hepatotoxicity include the following: prior autoimmune disease [27], prior immune-related adverse events from ICIs [28], high dose of ICIs, and the combination of ipilimumab and nivolumab [16]. In the present study, a logistic regression analysis showed that the use of anti-CTLA-4 inhibitor was an independent risk factor for ≥ grade 3 hepatotoxicity, and that a female sex and melanoma were confounding factors. Because anti-CTLA-4 inhibitor is associated with hepatitis, specific predictors of irSC factors could not be detected in this study.

This study had several limitations. First, the total number of patients with irSC or irHepatitis was small because of a lack of CT, and the unclassified hepatotoxicity remained in some cases. Therefore, a statistical analysis was not done for comparison between irSC and irHepatitis. The reason for the lack of CT data is that a standardized diagnostic strategy for irSC does not yet exist. According to the current guidelines for immunotherapy, all immune-related hepatotoxicity will be regarded as irHepatitis. Therefore, we propose that patients with a ≥ grade 2 ALP elevation that cannot be ascribed to other causes should be considered for close follow-up and radiological examination for the diagnosis of irSC. Early detection of irSC will lead to ICI discontinuation and/or initiation of corticosteroid therapy. Second, there is a selection bias in patients who underwent CT scans. In this study, CT scans were performed in 85% (11/13) of patients with a ≥ grade 3 AST or ALT elevations, while 3.6% (1/28) of patients with a grade 2 elevation. However, it is grade 3 or higher toxicities that requires immediate initiation of corticosteroids. Third, hepatotoxicity was graded by evaluating the AST/ALT values using the CTCAE system, and not according to the international normalized ratio and the presence of ascites or encephalopathy using the Drug Induced Liver Injury Network severity index, which is suitable for evaluating the severity of hepatotoxicity [29]. The CTCAE system is usually used to evaluate hepatotoxicity in oncology clinical trials and in management guidelines for immune-related adverse events [4, 5, 12]. Future prospective studies are needed to confirm our findings.

In conclusion, immune-related hepatotoxicity includes both irHepatitis and irSC, which have different characteristics such as the class of ICI that has been used and the onset pattern. Distinguishing between irHepatitis and irSC can help to predict the steroid response. Clinicians should be aware that an ALP elevation resulting in a cholestatic pattern might indicate the emergence of irSC, although the current management strategy for immunotherapy is based only on the AST, ALT, and total bilirubin values. This issue should be evaluated and validated in a large-scale clinical study.
